# Dr. Cappe, on Angina Pectoris

**Published:** 1800-09

**Authors:** Robert Cappe

**Affiliations:** York


GENTLEMEN,

AS the public attention is now much intereſted by Dr. Parry's valuable Treatiſe on Angina Pectoris, an account of a caſe of that diſeaſe, in ſome reſpects remarkable, may not be unacceptable to you. It occurred in the year 1797; but Angina Pectoris is ſo infrequent, that I have had no other opportunity of making trial of the medicine, which ſeemed, in this inſtance, highly beneficial; nor of determining, whether any credit be due to my conjectures on the nature of the diſeaſe. Still, it may not be without uſe, to relate a ſingle inſtance, in which this rare but fatal complaint was removed by means not employed before.

I believe I was not miſtaken, in thinking this caſe an inſtance of the diſeaſe, of which Dr. Heberden gave the firſt deſcription; but that may be determined, and the reaſon for the treatment I adopted may beſt be explained, by tranſcribing part of the notes which I wrote at the time.

Dec. 6, 1797. John Maynard, æt. 61, a carver in wood, was born in Berlin; has lived in London thirty-nine years, and enjoyed extraordinary good health till laſt winter. He is tall, well proportioned, thin, but pretty ſtout for his years, though a little bent forward, probably from ſtooping over his work. His anſwer to every queſtion, though a foreigner, was ſo diſtinct and preciſe, that I could not doubt the accuracy of the account he gave of his illneſs.

"Laſt March he became a patient at the Public Diſpenſary in Carey Street, London, having a cough, ſpitting of blood, and pain in his arms, near the inſertion of the deltoid muſcles, and down his fore arms, in the courſe of the muſculo-cutaneous nerves. He had been ill ſome time, but in about three months he was recovered; he left the Diſpenſary, and had no return of ſpitting of blood."

On the 6th of December he again became a patient at the Diſpenſary, and was committed to my care, by my friend Dr. Willan,

Willan.—"He has suffered much for about a month, from fits of "gasping for breath,"* occasioned by walking, efforts at stool, and bending forwards; but especially by stooping to buckle his shoes; even very slight exertions, as crossing his legs while sitting, will bring them on. In these fits he has an inexpressible sensation of severe pinching at the pit of his stomach, extending upwards, and a gasping for breath, which oblige him instantly to desist from all exertion. On pressing on the right side, below the ribs, near the scrobiculus cordis, he feels pain: this is the seat of his peculiar sensation.† He complains of pain in his loins, and in the iliac regions on bending his body. He has a constant cough, with very little expectoration. The peculiar sensations just described, have never seized him during a fit of coughing, but they have sometimes followed that effort. He never has had the sensation of his heart ceasing to beat; but on recovering from a fit, he feels it "flutter:"‡ It cannot be felt in any part of the thorax. Since these complaints began, he has had no pain in his arms."

"He finds his disease increasing: he now is never a whole day without fits, and sometimes has many in a day; they come on even when he is in bed, but are not more severe at night than in the day. He is habitually costive, having had a motion of the bowels only about once a week, for some years."

"His pulse was slow, and very weak; but so little remarkable, that, having felt it for a short time, I did not count it by my watch."

"He has no symptoms which indicate with certainty, an organic disease of the heart; though his age leaves little doubt that the arteries, and perhaps the valves of the heart, are not in their natural state. If there be no other disease, it is probable that the elasticity of the aorta is impaired; for this artery becomes less and less elastic, as old age advances. The valves at the origin of the aorta, in consequence of this change, perform their office imperfectly;‖ some part of the blood returns into the heart, and the impulse of the heart on the blood in circulation is diminished."

"The

---

* This is his own expression. I never saw him during a paroxysm.

† Dr. Heberden says, the pain always inclines more to the left side. See Med. Trans. Vol. II. p. 63.

‡ This word was used by the patient; but he did not mean to express by it, a palpitation of the heart;—he never had palpitation of the heart.

‖ These valves not being fully extended, the blood will be impeded in its entrance into the coronary arteries.

"The ſymptoms of true* Angina Pectoris, in which no change of ſtructure in the heart or its veſſels, is diſcovered in diſſection, may, perhaps, depend on defect of force in the heart. It is probable, that in ſome inſtances that organ becomes abſolutely weaker; as it has been found on diſſection, that the fibres had loſt their tenacity in an extraordinary degree: In other inſtances, the muſcles may not have loſt their vigour; but the obſtacles to be overcome may have been increaſed."

"Theſe conjectures reſpecting the nature of the diſeaſe, ſeem not improbable; for ſymptoms reſembling Angina Pectoris accompany ſome organic diſeaſes, in which the heart cannot communicate to the whole maſs of blood, an impulſe equal to that which it imparts in a ſound ſtate."

"In Maynard's caſe, I ſuſpect that the power of the heart is diminiſhed, as its motion cannot be felt, and the pulſe is very feeble; I therefore wiſh to try the effect of *argentum nitratum*, for that ſubſtance has a very remarkable influence both on the nerves and the muſcles, even when their communication with the brain is deſtroyed, and their ſupply of blood cut off; and it has been, in ſome inſtances, an effectual remedy in epilepſy, a convulſive diſeaſe ſubject to irregular paroxyſms.—In the year 1793, in making experiments with metals on the irritability of the muſcles, both of cold-blooded and warm-blooded animals, I found that the muſcles of a detached limb were rendered more vigorous;† and when they had ceaſed to be convulſed on the application of metals, they were reſtored to energy by touching a ſingle point of the nerve with *argentum nitratum*. In other experiments, I laid a frog, with its head cut off, and abdomen opened, upon a plate of zinc, and endeavoured to excite convulſions, by making a communication between the viſcera and the plate of zinc by a ſilver probe; but in vain, till I had touched the peritonæum with argentum nitratum. After this application, violent convulſions of the lower limbs were excited, by making a commu-

* This was written under the impreſſion, that the name Angina Pectoris had been applied, in ſome inſtances, to diſeaſes which did not correſpond with the diſeaſe to which Dr. Heberden gave that name, either in ſymptoms, or appearance on diſſection; but in other inſtances, though the ſymptoms were the ſame as thoſe deſcribed by Dr. Heberden, the appearance on diſſection was very different. He diſcovered no organic diſeaſe. See Med. Trans. Vol. III. p. 8.

† I uniformly found the very oppoſite effect from *kali purum*; and I in vain endeavoured to reſtore the energy of the muſcles, by the ſubſequent application of *argentum nitratum*, diluted *ſulphuric acid*, and *diſtilled vinegar*.

a communication again between the viſcera and the metals. Hence it appears, that argentum nitratum applied to a ſingle joint of the nervous ſyſtem, produces ſome change through the whole body of the animal; and that effect ſeems conducive to muſcular vigour."

"I was led by theſe facts, to preſcribe argentum nitratum to Maynard; for it is probable, that taken into the ſtomach, it muſt come in contact with a ſurface, not leſs furniſhed with nerves than the peritonæum. That ſubſtances taken into the ſtomach, produce effect through the medium of the nerves, cannot be doubted, ſince emetics* do not occaſion vomitting when the *par vagum* is divided."

"After what length of time the effect of argentum nitratum ceaſes, I do not know; I never made that an object of *attention* in my experiments; but, from accidental obſervation, I know that its influence is in a ſhort time diminiſhed, and within twenty-four hours has entirely ceaſed: though the ſame effect may again be produced, by again applying the *argentum nitratum* to the nerve. This I have found ſucceed each day for ten days, after the leg of a frog had been detached from the body, from which the head had been previouſly cut off."

"Maynard was ordered to take a quarter of a grain of argentum nitratum, three times a day. He was deſired to uſe aloetic pills, when neceſſary, to regulate the ſtate of his bowels.

"Dec. 8th. The patient ſtanding; his pulſe intermits often, and at unequal intervals: it beats at the rate of 68 in a minute. He is upon the whole, much better. The fits are not ſo eaſily excited, are not ſo frequent, and do not laſt ſo long. Let him continue the argentum nitratum."†

"Dec. 11th. The fits are leſs frequent, and leſs ſevere. His pulſe is about 69: It does not intermit; but, many times in a minute, a beat is perceived much leſs forcible than the reſt."

"Dec. 12th. His pulſe is 67: It does not intermit; but ſome beats are leſs forcible than the reſt: His bowels are regular. On the whole, he is much better."

"Dec. 15th. There is no alteration in his complaints, nor in his pulſe. He was not quite ſo well yeſterday; but he had been coſtive two days, and was relieved after an evacuation: He ſtill complains of pain a little above the left hip, when he bends forwards. He has not quite ſo much pain in his loins."

"Dec.

* See Dr. Haighton's Exper. Med. Soc. Lond. Vol. II.

† This medicine was not omitted till his recovery was eſtabliſhed.

"Dec. 18th. He has been coftive fince the 15th. On the 17th, he had pain and fluttering of his heart; to-day he is better. His pulfe is 64; it does not intermit, but its force ftill remits: He has more pain in his loins. Let him ufe the volatile liniment."

"Dec. 20th. His pulfe is 64, ftrong, and without remiffion. He had two fits yefterday, not occafioned by any great exertion: his bowels are regular. To-day he had a fit after he reached the Difpenfary; he had walked more than half a mile. The pain in his loins feems moving downward: He had no pinching at the fcrobiculus cordis, in the fits either of yefterday or to-day; but he had "fluttering" and pain, begining about the middle of the fternum; it moved acrofs the left fide: the gafping for breath was not very urgent. Let him take argentum nitratum four times a day."

"Dec. 29th. His pulfe is 68, with one intermiffion in a minute. He had a flight fit on going up ftairs: He has much lefs pain."

"Jan. 8th. He has pain in his right fide, and the calves of his legs: the pain is nearly removed from his left hip. He has had no fit fince the laft report: His bowels are at prefent coftive: His pulfe is 64, regular and ftronger. He looks much better, and fatter in the face."

"Jan. 15th. He is much better; he had a fit on the 13th. His pulfe is 64, and regular."

"Jan. 19th. He has had no fit fince the 13th: His pulfe is 61, and regular. Let him continue the argentum nitratum; and take a fcruple of Peruvian bark three times a day."

"Jan. 24th. His pulfe is 69, regular, and firmer, but not full. He has lefs pain in his back: the bark keeps his bowels open. Let the medicines be continued."

"Feb. 9th. He is much better. After this time he had very few, and thofe only flight returns of fits. His urine was never copious."

About the middle of February I left London; but I often heard of Maynard; and in October, 1797, I heard that he had continued free from his former complaints, and was pretty well; but was obliged, occafionally, to take aloetic pills.

I have tranfcribed each day's report, to fhew the progrefs of the cafe. The remiffion of the fymptoms from the time when he began to take argentum nitratum, though the difeafe was daily growing worfe, leaves no doubt that the recovery fhould be attributed to this medicine: It produced no fenfible effect; it did not even keep the bowels regular, though I have fometimes found it occafion diarrhoea. That is the only effect I ever ever obſerved from argentum nitratum in many different diſorders, in ſome of which it proved a ſuccesſful remedy.

I have ſtated my conjectures on the nature of the diſeaſe, as they occurred; and I think them corroborated by the facts that Dr. Parry has lately publiſhed. He relates, that in ſeveral inſtances of Angina Pectoris, the coronary arteries, the veſſels that carry the blood for the ſupport of the heart, were oſſified; and in ſome inſtances, their capacity was diminiſhed by a membranous tube, formed within them.* Theſe, or any diſeaſes, which may diminiſh the quantity of arterial blood diſtributed through the heart, will probably impair the muſcular force of that organ; for all other muſcles loſe their power when the ſupply of arterial blood is diminiſhed.

Fourcroy, with ſome of his aſſociates in the Academy of Sciences, proved by accurate experiments, that the quantity of oxygen abſorbed in reſpiration, is increaſed in proportion to the increaſe of muſcular exertion: the frequency of the pulſe is alſo increaſed. And Anatomiſts obſerve, that the quantity of blood diſtributed through muſcles, is in proportion to the exertions to which the muſcles have been accuſtomed. The colour is higher, and the blood veſſels are larger, in thoſe muſcles which have been accuſtomed to much exertion, than in correſponding muſcles in other bodies, which have been leſs exerted. From theſe facts, it is evident, that the blood veſſels do not contain more arterialized blood, than the muſcles require for ordinary exertions; and therefore, when the quantity is diminiſhed, the muſcles muſt be unequal to their higher exertions. The heart, therefore, when its coronary veſſels are diſeaſed, will not be able to propel the blood when returned upon it in greater quantity, from exertions of the body, or affections of the mind; nor when the obſtacles to the motion of the blood are increaſed by the poſture of the body.

From the ſymptoms of Angina Pectoris, and from diſſection, it ſeems probable, that this diſeaſe ariſes from a defect of force of the heart, either abſolute or relative.

The blood, in conſequence of ſuch diſeaſe in the heart, is leſs quickly ſubmitted to the influence of the air in the lungs; hence follows a ſenſe of ſuffocation, which gives riſe to more frequent efforts of breathing, to gaſping, and deep ſighs. And the heart perhaps, from the diſproportioned effort, may

* See Inquiry into the Symptoms and Cauſes of Syncope Anginoſa, p. 4, 12, 24, 33.

may be feized with cramp, as other mufcles are feized with cramp in great, and efpecially in protracted, efforts.

The cafe I have related, leads to many other obfervations; but I have intruded too far on your Journal, to add more than that I am,

GENTLEMEN,

With great refpect, your obedient fervant,

*York, Feb.* 20, 1800. ROBERT CAPPE.

P. S. Thefe notes were fent to a friend in London, in whofe judgment I have great confidence, with a requeft that he would tranfmit them to the Editors of the Medical and Phyfical Journal, if he thought they might be worthy of their attention. My friend expreffed a doubt, whether the cafe was really Angina Pectoris, and fuggefted a change of title for the paper. I fhould acquiefce in his judgment, and adopt the change, if the cafe had not been confidered by fome friends who faw it, as Angina Pectoris; and the notes, marked with inverted commas, had not been written at the time on this fuppofition. I have, therefore, to requeft of you, gentlemen, to accept them, fuch as they are; and I am not without hope, that the cafe, which ftrongly refembles Angina Pectoris, may not be found uninterefting by thofe who would affign it fome other name.

---

### *To the Editors of the Medical and Phyfical Journal.*

GENTLEMEN,

